# CD4 Cell Count Threshold for Cryptococcal Antigen Screening of HIV-Infected Individuals: A Systematic Review and Meta-analysis

**DOI:** 10.1093/cid/cix1143

**Published:** 2018-03-04

**Authors:** Nathan Ford, Zara Shubber, Joseph N Jarvis, Tom Chiller, Greg Greene, Chantal Migone, Marco Vitoria, Meg Doherty, Graeme Meintjes

**Affiliations:** 1 HIV Department, World Health Organization, Geneva, Switzerland; 2 Department of Infectious Disease Epidemiology, Imperial College London, United Kingdom; 3 Botswana-UPenn Partnership, Gaborone, Botswana; 4 Botswana Harvard AIDS Institute Partnership, Gaborone, Botswana; 5 Department of Clinical Research, Faculty of Infectious and Tropical Diseases, London School of Hygiene and Tropical Medicine, United Kingdom; 6 Mycotic Diseases Branch, Centers for Disease Control and Prevention, Atlanta, Georgia; 7 Wellcome Trust Centre for Infectious Diseases Research in Africa, Institute of Infectious Disease and Molecular Medicine South Africa; 8 Department of Medicine, Faculty of Health Sciences, University of Cape Town, South Africa

**Keywords:** advanced HIV disease, CrAg, cryptococcal antigen, cryptococcal meningitis, HIV

## Abstract

**Background:**

Current guidelines recommend screening all people living with human immunodeficiency virus (PLHIV) who have a CD4 count ≤100 cells/µL for cryptococcal antigen (CrAg) to identify those patients who could benefit from preemptive fluconazole treatment prior to the onset of meningitis. We conducted a systematic review to assess the prevalence of CrAg positivity at different CD4 cell counts.

**Methods:**

We searched 4 databases and abstracts from 3 conferences up to 1 September 2017 for studies reporting prevalence of CrAg positivity according to CD4 cell count strata. Prevalence estimates were pooled using random effects models.

**Results:**

Sixty studies met our inclusion criteria. The pooled prevalence of cryptococcal antigenemia was 6.5% (95% confidence interval [CI], 5.7%–7.3%; 54 studies) among patients with CD4 count ≤100 cells/µL and 2.0% (95% CI, 1.2%–2.7%; 21 studies) among patients with CD4 count 101–200 cells/µL. Twenty-one studies provided sufficient information to compare CrAg prevalence per strata; overall, 18.6% (95% CI, 15.4%–22.2%) of the CrAg-positive cases identified at ≤200 cells/µL (n = 11823) were identified among individuals with a CD4 count 101–200 cells/µL. CrAg prevalence was higher among inpatients (9.8% [95% CI, 4.0%–15.5%]) compared with outpatients (6.3% [95% CI, 5.3%–7.4%]).

**Conclusions:**

The findings of this review support current recommendations to screen all PLHIV who have a CD4 count ≤100 cells/µL for CrAg and suggest that screening may be considered at CD4 cell count ≤200 cells/µL.

The burden of cryptococcal meningitis among people living with human immunodeficiency virus (PLHIV) remains substantial despite scale-up of antiretroviral therapy (ART) [1]. A recent review estimated that globally there were 223 100 incident cryptococcal meningitis cases (with 73% of the cases occurring in sub-Saharan Africa), resulting in almost 200000 deaths in 2014 [2].

Current World Health Organization (WHO) guidelines recommend screening all PLHIV who have a CD4 count ≤100 cells/µL for cryptococcal antigen (CrAg) to identify those patients with cryptococcal disease who could benefit from preemptive fluconazole treatment prior to the onset of meningitis. CrAg may be detected several weeks before clinical features of cryptococcal meningitis become apparent [3]. Providing preemptive fluconazole treatment during this period of antigenemia prior to onset of meningitis symptoms has been found to be life saving and cost effective across a range of settings [4–7]. Some countries have chosen higher CD4 cell count thresholds for their cryptococcal screening guidelines: Ethiopia has adopted a cutoff of 150 cells/µL, whereas in Rwanda CrAg screening is done at a CD4 count of ≤200 cells/µL.

Recent WHO guidelines advise that a CD4 threshold of ≤200 cells/µL be used to define patients who have advanced HIV disease [8], and studies have suggested there may be benefit to CrAg screening among PLHIV using a higher CD4 count threshold of ≤200 cells/µL to identify additional numbers of PLHIV at risk of developing cryptococcal meningitis [9–12]. The current recommended threshold for CrAg screening at CD4 count ≤100 cells/µL is based on evidence from a limited number of studies, and there may be benefits to simplifying screening strategies to target all patients with advanced human immunodeficiency virus (HIV) disease. We conducted a systematic review to assess prevalence of CrAg positivity at CD4 count ≤100 cells/µL compared to 101–200 cells/µL across a range of settings.

## METHODS

### Search Strategy and Selection Criteria

This systematic review was conducted according to the Preferred Reporting Items for Systematic Reviews and Meta-Analyses (PRISMA) guidelines [13]. Using a study protocol (available from the corresponding author), we sought randomized and quasi-randomized controlled trials, and comparative and noncomparative observational studies reporting prevalence of CrAg positivity according to CD4 cell count strata.

Using a broad search strategy combining terms for HIV infection and CrAg screening, 3 investigators (N. F., Z. S., C. M.), working independently and in duplicate, screened titles and abstracts from Medline via PubMed, Embase, and the Cochrane library, from inception to 1 September 2017. Abstracts from the International AIDS Society conferences, the Conferences on Retroviruses and Opportunistic Infections, and the International Conference on Cryptococcus and Cryptococcosis were also screened from 2012 to 2017 to identify studies that have been recently completed but not yet published in full. Database searches were supplemented by screening bibliographies of review articles and all included full-text articles. The same investigators scanned all abstracts and full-text articles and achieved consensus on final study inclusions.

Reasons for exclusions included studies using samples other than serum, plasma, or whole blood. If studies included patients with a history of cryptococcal disease or overt clinical meningitis, then these patients were excluded from the study denominators and numerators included in this review; where it was not possible to remove these patients from the study population, the studies were only included if <10% of patients met these criteria. No language or geographical restrictions were applied.

### Data Extraction

The same 3 investigators extracted data following a predefined protocol and using a standardized and piloted extraction form. Study characteristics included design, year, population, location, ART status, CrAg positivity by CD4 cell count stratum, and active tuberculosis (TB) infection. Where reported, outcomes for CrAg-positive patients who received or did not receive fluconazole were also extracted. Additional information was extracted to inform an assessment of risk of bias and the certainty of the evidence using the *Grading* of Recommendations Assessment, Development, and Evaluation (GRADE) approach [14].

### Statistical Analysis

To estimate CrAg prevalence by CD4 cell count stratum, point estimates and corresponding 95% confidence intervals (CIs) were calculated and data were pooled using random-effects meta-analysis [15], following data transformation [16, 17]. The same approach was used to summarize clinical outcomes among CrAg-positive patients started and not started on fluconazole. Prevalence odds ratios were calculated to compare diagnostic yield by CD4 cell count strata (≤100 vs 101–200 cells/µL) using random effects models. Heterogeneity was assessed though visual inspection of forest plots and subgroup analyses to examine potential differences by geographical region, clinical setting, type of CrAg screening test used, and sample type. We analyzed all data with Stata version 13.0 software.

## RESULTS

### Characteristics of Included Studies

**Figure 1.**
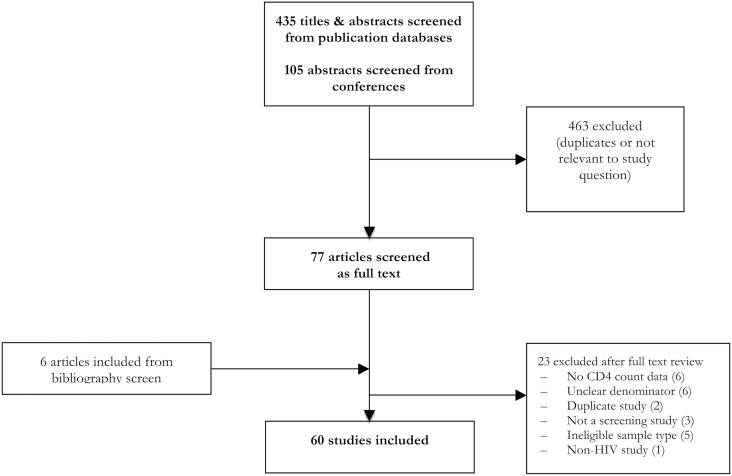
Study selection process. Abbreviation: HIV, human immunodeficiency virus.

From an initial screen of 540 titles, 60 studies were included in this review (Figure 1) [6, 9–12, 18–70]. Among these, 40 were prospective studies (including one randomized trial) and 20 were retrospective studies; 42 studies were published in full, 16 were abstracts, and additional unpublished data were provided from Médecins Sans Frontières (MSF)–supported HIV programs in Kenya and the Democratic Republic of Congo. Data came from 28 countries, with the majority of studies (41) carried out in Africa. Median age of patients ranged from 30 to 47 years, and the proportion who were female ranged from 20% to 74%. Date of study end ranged from 2013–2017 (median 2014). Most studies (41 studies [66%]) used sera as the sample type and a lateral flow assay (34 studies [57%]). Thirty-two studies reported that all patients screened (n = 18657) were ART naive, while 16 studies (n = 6950) reported that a proportion of patients were ART experienced (median, 41.7% [interquartile range], 18.4%–72.4%) (Supplementary Appendix).

Risk of bias overall was assessed as being moderate (Supplementary Appendix). The majority of studies used a prospective study design (40 studies), were published in full (42 studies), and had <10% missing data (52 studies). Only 2 studies reported blinding of investigators and only 3 studies reported random sampling of patients. In subgroup analysis, none of these risk of bias indicators influenced CrAg prevalence estimates. Overall, the certainty of evidence was rated as moderate.

### Prevalence of Cryptococcal Antigenemia

**Figure 2.**
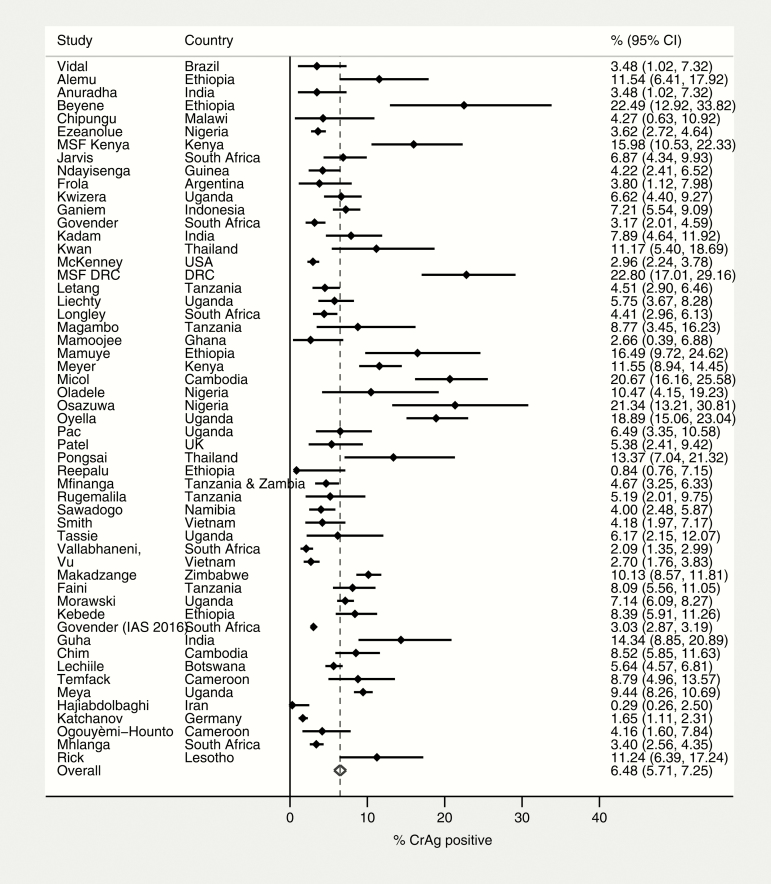
Prevalence of CrAg positivity among patients with CD4 count ≤100 cells/μL. Abbreviations: CI, confidence interval; CrAg, cryptococcal antigen; DRC, Democratic Republic of the Congo; IAS, International AIDS Society; MSF, Médecins Sans Frontières.

**Figure 3.**
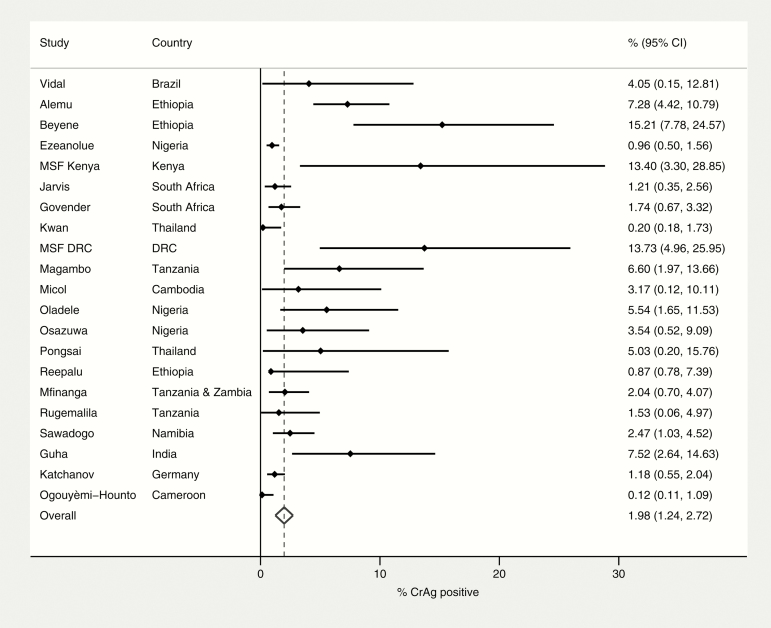
Prevalence of CrAg positivity among patients with CD4 count 100–200 cells/μL. Abbreviations: CI, confidence interval; CrAg, cryptococcal antigen; DRC, Democratic Republic of the Congo; MSF, Médecins Sans Frontières.

The pooled prevalence of cryptococcal antigenemia as determined by CrAg positivity was 6.5% (95% CI, 5.7%–7.3%; 54 studies) among patients with CD4 count ≤100 cells/µL and 2.0% (95% CI, 1.2%–2.7%; 21 studies) among patients with CD4 count 101–200 cells/µL (Figures 2 and 3).

**Figure 4.**
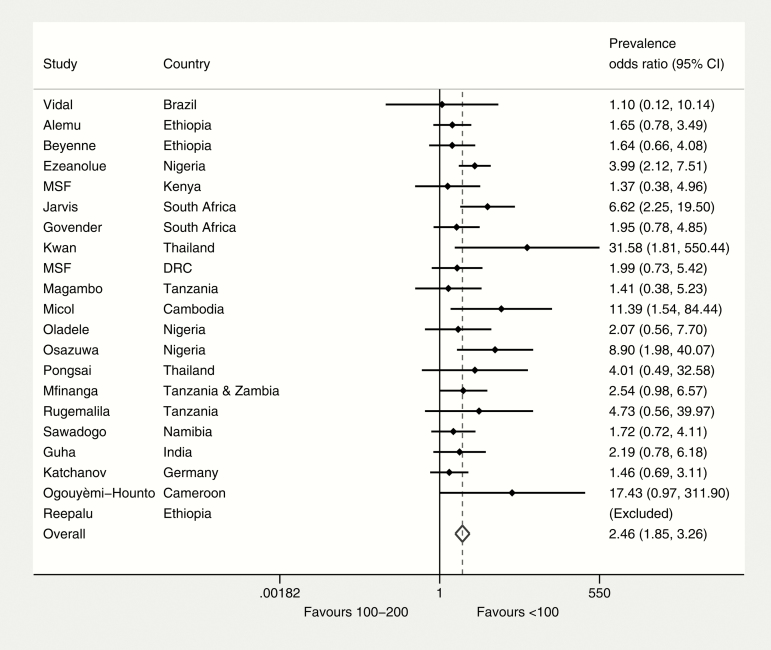
CrAg prevalence odds ratio (CD4 count ≤100 cells/μL vs 100–200 cells/μL). Abbreviations: CI, confidence interval; CrAg, cryptococcal antigen; DRC, Democratic Republic of the Congo; MSF, Médecins Sans Frontières.

Twenty-one studies provided sufficient information to compare CrAg prevalence at CD4 count ≤100 cells/µL vs 101–200 cells/µL within each study. The prevalence odds ratio comparing CD4 count ≤100 cells/µL and CD4 count 101–200 cells/µL was 2.5 (95% CI, 1.9–3.3) (Figure 4). Overall, 18.6% (95% CI, 15.4%–22.2%) of the total CrAg-positive cases identified in this sample of patients with CD4 ≤200 cells/µL (n = 11823) were among individuals with a CD4 count 101–200 cells/µL.

**Figure 5.**
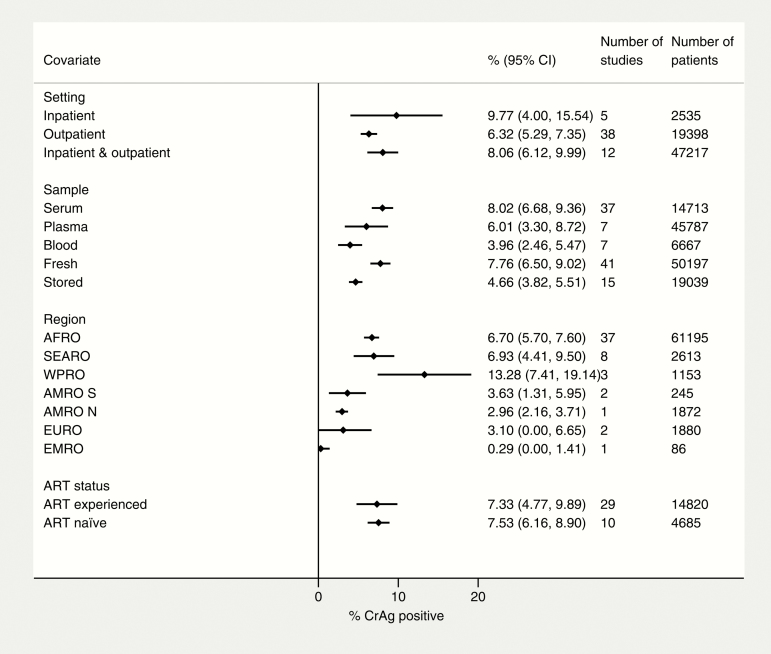
Factors associated with CrAg positivity at CD4 count ≤100 cells/μL. Abbreviations: ART, antiretroviral therapy; AFRO, Africa Region; AMRO N, North America Region; AMRO S, South America Region; CI, confidence interval; CrAg, cryptococcal antigen; EMRO, Mediterranean Region; EURO, Europe Region; SEARO, South-East Asia Region; WPRO, Western Pacific Region.

Among patients with CD4 count ≤100 cells/µL, CrAg positivity ranged from 0.3% (95% CI, 0.3%–2.5%) in Iran to 22.8% (95% CI, 17%–29.2%) in DRC (MSF, unpublished data). In subgroup analysis, there was substantial variability by geographical region, with CrAg prevalence highest in the Africa Region (6.7% [95% CI, 5.7%–7.6%]), the South-East Asia Region (6.9% [95% CI, 4.4%–9.5%]), and the Western Pacific Region (13.3% [95% CI, 7.4%–19.1%]). CrAg prevalence was also higher among inpatients (9.8% [95% CI, 4.0%–15.5%]) than outpatients (6.3% [95% CI, 5.3%–7.4%]). A higher prevalence was also seen in studies that used nonfrozen samples (7.8% [95% CI, 6.5%–9.0%]) rather than stored samples (4.7% [95% CI, 3.8%–5.5%]). CrAg prevalence was similar comparing studies that only enrolled ART-naive patients and those that included both ART-naive and ART-experienced patients (Figure 5).

### Clinical Outcomes

Nineteen studies reported outcomes among 353 CrAg-positive, asymptomatic PLHIV who were started on fluconazole prophylaxis [9, 11, 19, 21, 24–27, 31, 38, 42, 52, 54, 55, 60–62, 64, 70], with clinical outcomes from one study [19] reported in a separate report [71]. Median follow-up time was 9 months (interquartile range, 6–12 months). Of these, 34 (9.6%) of patients had died, among whom none were documented to have died of cryptococcal meningitis. Nineteen (5.4%) developed incident cryptococcal disease. Fourteen studies reported outcomes among 118 CrAg-positive, asymptomatic PLHIV who were not started on fluconazole prophylaxis [9, 11, 12, 20, 21, 25–27, 31, 38, 41, 42, 70, 71]. Of these, 22 (18.6%) had died, among whom 2 were documented to have died of cryptococcal meningitis; 3 others developed incident cryptococcal disease.

Thirteen studies reported the prevalence of TB disease among patients who were CrAg positive, using different TB screening approaches [9, 11, 19–22, 26, 28, 33, 41, 46, 57, 72]. Among 234 patients screened CrAg positive, 45 also were diagnosed with TB, giving an overall prevalence of coexistent disease of 19.2% (95% CI, 14.4%–24.9%).

## DISCUSSION

Cryptococcal meningitis remains an important cause of morbidity and mortality among people with HIV, despite major improvements in access to HIV testing and treatment services [2]. This is largely explained by an enduring burden of advanced HIV disease, either because people present late for diagnosis and care or, increasingly, because PLHIV interrupt ART for a period during which time their CD4 cell count drops, placing them at risk of major opportunistic infections including cryptococcal meningitis [73].

This review estimated prevalence from available studies, by country and region, and found a high prevalence of CrAg positivity among people with advanced HIV disease that, consistent with expectations, was higher among those with a lower CD4 cell count. These findings support current guidance to screen all individuals presenting for care with a CD4 count ≤100 cells/µL. Prevalence at CD4 count ≤100 cells/µL was highest in the Africa, South-East Asia, and Western Pacific regions. The finding that one fifth of CrAg-positive patients were also found to have TB supports the inclusion of CrAg and TB testing as part of a package to manage advanced HIV disease.

This review further suggests that there may be additional benefit to screening individuals at CD4 cell count up to 200 cells/µL, depending on availability of resources and considering the practical advantage of providing the same package of care to all patients with advanced HIV disease within a public health approach. Almost one-fifth of CrAg-positive cases identified at CD4 count ≤200 cells/µL are identified at CD4 between 101 and 200 cells/µL. Cost-effectiveness analyses have so far focused on the benefit of CrAg screening at CD4 count ≤100 cells/µL, and there is some evidence of benefit down to a prevalence of 0.6% of cryptococcal antigenemia [4]. Further cost-effectiveness research is needed to assess the value of screening at a higher CD4 cell count threshold of 200 cells/µL, which has already been suggested to be cost saving if carried out in inpatient settings [9].

There is a growing evidence base supporting the clinical benefit and cost effectiveness of CrAg screening in combination with enhanced ART adherence and delivery interventions. A trial conducted in the United Republic of Tanzania and Zambia randomized 1999 ART-naive adults living with HIV with a CD4 count <200 cells/µL to receive enhanced clinic-based care with CrAg screening and preemptive antifungal treatment for those who were CrAg positive; importantly, additional community support including ART delivery and adherence counseling was provided to the intervention group. The trial reported a 28% reduction in mortality (13% vs 18%) among people receiving the intervention compared to standard care [52]. In an unpublished post hoc analysis, a statistically significant mortality reduction was found in both people with a CD4 count <100 cells/µL (mortality rate ratio, 0.75 [95% CI, .58–.95]) and those with a CD4 cell count of 101–200 cells/µL (mortality rate ratio, 0.56 [95% CI, .32–.97]).

A recent study from South Africa reported the numbers of patients starting ART in 2016 at different CD4 cell count thresholds using data from the national laboratory database [74]. According to this analysis, 128888 patients (16.8% of the total) started ART at a CD4 count ≤100 cells/µL and 123 164 (16.1%) started at a CD4 count of 101–200 cells/µL. Applying the pooled prevalence estimates from this review, 8249 patients (95% CI, 7347–9280) would theoretically be identified as being CrAg positive at a CD4 screening threshold of 100 cells/µL, and an additional 2463 patients (95% CI, 1478–3325) would be identified if a threshold of CD4 200 cells/µL were applied.

The majority of studies included in this review were carried out in Africa, and the findings of this review are of greatest relevance to settings with a high burden of HIV and cryptococcal meningitis. Nevertheless, cryptococcal disease remains an important cause of illness and death among people with HIV in high-income settings, and the role of CrAg screening and preemptive therapy should be considered in these settings [48].

Strengths of this review include a broad and inclusive search strategy that allowed for the identification of a large number of studies for analysis. Heterogeneity was anticipated, and explored using standard methods that increased confidence in the overall findings. The main limitations to note are the limited reporting of important information which may influence CrAg prevalence, notably ART experience, which was missing for one-fifth of studies included in this review, and the limited reporting of clinical outcomes. Another limitation relates to methodological quality, with a number of studies being carried out retrospectively and only 3 studies reporting random patient sampling. While methodological quality did not appear to importantly influence the prevalence estimates, future studies are encouraged to take steps to improve methodological rigor.

This review highlights several directions for research, including the cost effectiveness of screening at higher CD4 cell counts and the appropriateness and cost effectiveness of screening ART-experienced adults and adolescents with low CD4 counts.

In conclusion, the findings of this review support current recommendations to screen all adults and adolescents who have a CD4 count ≤100 cells/µL for CrAg, whether they are ART naive or experienced, and provide preemptive fluconazole treatment to those testing positive. Consideration should also be given to screening at a higher CD4 count of ≤200 cells/µL in settings where there are sufficient resources to implement such an approach, or where a simplified package of care for advanced disease is required based on a unified CD4 threshold.

## Supplementary Data

Supplementary materials are available at *Clinical Infectious Diseases* online. Consisting of data provided by the authors to benefit the reader, the posted materials are not copyedited and are the sole responsibility of the authors, so questions or comments should be addressed to the corresponding author.

Supplementary Table

Supplementary Table Study Characteristics

## Notes

***Acknowledgments.*** We thank Sayoki Mfinanga and Shabbar Jaffar for providing mortality reduction data by CD4 count threshold from the REMSTART trial, and David Maman for providing unpublished data from MSF programmes.

***Financial support.*** This work was supported by several sources of funding to the HIV Department, mainly funding from the US President’s Emergency Plan for AIDS Relief through the US CDC cooperative agreement and the Bill & Melinda Gates Foundation. G. M. is supported by the Wellcome Trust (grant number 098316) and the South African Research Chairs Initiative of the Department of Science and Technology and National Research Foundation of South Africa (grant number 64787). J. N. J. is supported by the Penn Center for AIDS Research, a National Institutes of Health–funded program (grant number P30 AI 045008). This Supplement was supported by funds from the Bill & Melinda Gates Foundation.

***Supplement sponsorship.*** This article appears as part of the supplement “Advanced HIV Disease,” sponsored by the World Health Organization.

***Potential conflicts of interest.*** All authors: No reported conflicts of interest. All authors have submitted the ICMJE Form for Disclosure of Potential Conflicts of Interest. Conflicts that the editors consider relevant to the content of the manuscript have been disclosed.
