# Supplementary material for: CD4 Cell Count Threshold for Cryptococcal Antigen Screening of HIV-Infected Individuals: A Systematic Review and Meta-analysis
Source: Clin Infect Dis. 2018 Mar 4;66(Suppl 2):S152–9. doi: 10.1093/cid/cix1143 (PMC5850628; doi:10.1093/cid/cix1143)
Supplement: Supplementary Table [file cix1143_suppl_supplementary_table.docx]

| **Study** | **Prospective design** | **Random sampling** | **Blinding** | **Missing data<10%** | **Published in full** |
| --- | --- | --- | --- | --- | --- |
| Ake | Y | N | N | Y | N |
| Alemu | Y | N | N | Y | Y |
| Andama | Y | N | Y | Y | Y |
| Anuradha | Y | N | N | Y | Y |
| Bedelle | Y | N | N | Y | Y |
| Beyene | Y | N | N | Y | Y |
| Chim | N | N | N | N | Y |
| Chipungu | Y | N | N | Y | Y |
| Ezeanolue | N | N | N | Y | Y |
| Faini | Y | N | N | Y | N |
| Frola | Y | N | N | Y | Y |
| Ganiem | Y | N | N | Y | Y |
| Gonzales | N | N | N | Y | N |
| Govender | Y | N | N | Y | Y |
| Govender (IAS 2016) | N | N | N | Y | N |
| Guha | Y | N | N | Y | N |
| Hajiabdolbaghi | Y | N | N | Y | Y |
| Jarvis | N | N | N | Y | Y |
| Kadam | Y | N | N | Y | Y |
| Katchanov | N | N | N | Y | Y |
| Kebede | Y | N | N | Y | N |
| Kwan | N | N | N | Y | Y |
| Kwizera | Y | N | N | Y | Y |
| Lechiile | Y | N | N | N | N |
| Letang | Y | N | N | Y | Y |
| Liechty | N | N | N | Y | Y |
| Longley | Y | N | N | Y | Y |
| Luzinda | Y | N | N | Y | N |
| Magambo | Y | N | N | Y | Y |
| Makadzange | Y | N | N | Y | N |
| Mamoojee | N | N | N | N | Y |
| Mamuye | Y | N | N | Y | Y |
| McKenney | N | Y | N | Y | Y |
| Mendes | N | N | N | N | N |
| Meya | Y | N | N | Y | N |
| Meyer | N | N | N | N | Y |
| Mfinanga | Y | Y | N | Y | Y |
| Mhlanga | Y | N | N | Y | N |
| Micol | Y | N | N | Y | Y |
| Morawski | Y | Y | N | Y | N |
| MSF DRC | N | N | N | Y | N |
| MSF Kenya | N | N | N | Y | N |
| Ndayisenga | N | N | N | N | N |
| Ogouyèmi-Hounto | Y | N | N | Y | Y |
| Oladele | Y | N | N | Y | Y |
| Osazuwa | Y | N | N | Y | Y |
| Oyella | Y | N | N | Y | Y |
| Pac | Y | N | N | Y | Y |
| Patel | N | N | Y | Y | Y |
| Pongsai | N | N | N | N | N |
| Reepalu | Y | N | N | N | Y |
| Rick | Y | N | N | Y | Y |
| Rugemalila | Y | N | N | Y | Y |
| Sawadogo | Y | N | N | Y | Y |
| Smith | N | N | N | Y | Y |
| Tassie | Y | N | N | Y | Y |
| Temfack | Y | N | N | Y | N |
| Vallabhaneni, | N | N | N | N | Y |
| Vidal | Y | N | N | Y | Y |
| Vu | Y | N | N | Y | N |
