# Supplementary material for: CD4 Cell Count Threshold for Cryptococcal Antigen Screening of HIV-Infected Individuals: A Systematic Review and Meta-analysis
Source: Clin Infect Dis. 2018 Mar 4;66(Suppl 2):S152–9. doi: 10.1093/cid/cix1143 (PMC5850628; doi:10.1093/cid/cix1143)
Supplement: Supplementary Table Study Characteristics [file cix1143_suppl_supplementary_table_study_characteristics.docx]

| **Study** | **Country** | **Setting** | **Age** | **% Female** | **Median CD4 (cells/mm^3^)** | **% on ART** |
| --- | --- | --- | --- | --- | --- | --- |
| Ake^1^ | Uganda, Kenya, Tanzania, Nigeria | Outpatient | 40 | 59 | NR | NR |
| Alemu^2^ | Ethiopia | Outpatient | 36 | 56 | 123 | 74.0 |
| Andama^3^ | Uganda | Inpatient | 33 | 53 | 51 | 18.5 |
| Anuradha^4^ | India | In & outpatient | 36 | 21 | 54.9 | 0 |
| Bedell^5^ | Malawi | Outpatient | 36 | 59 | 129 | 0 |
| Beyene^6^ | Ethiopia | In & outpatient | 33 | 55 | 151-200 | 70.9 |
| Chim^7^ | Cambodia | In & outpatient | 38 | 46 | 28 | 0 |
| Chipungu^8^ | Malawi | Outpatient | 36 | 46.9 | 124 | 0 |
| Ezeanolue^9^ | Nigeria | Outpatient | 32 | 54 | NR | 0 |
| Faini^10^ | Tanzania | In & outpatient | 39 | 55 | 58 | 0 |
| Frola^11^ | Argentina | Outpatient | 38 | 31 | 46 | 23.9 |
| Ganiem^12^ | Indonesia | Outpatient | 30 | 24 | 20 | 0 |
| Gonzales^13^ | Colombia | NR | 40 | 34.6 | NR | 17.3 |
| Govender^14^ | South Africa | Outpatient | 38 | 62 | NR | NR |
| Govender^15^ | South Africa | In & outpatient | NR | NR | NR | NR |
| Guha^16^ | India | Outpatient | 38 | 20 | 78 | 0 |
| Hajiabdolbaghi^17^ | Iran | NR | 35.6 | 26 | 45 | NR |
| Jarvis^18^ | South Africa | Outpatient | 33 | 74 | 97 | 0 |
| Kadam^19^ | India | In & outpatient | 40 | 35 | 65 | 18.3 |
| Katchanov^20^ | Germany | Inpatient | 41 (CrAg+) | 13 (CrAg+) | 40 (CrAg+) | NR |
| Kebede^21^ | Ethiopia | Inpatient | NR | NR | NR | 0 |
| Kwan^22^ | Thailand | Outpatient | 31 | 100 | 147 | 0 |
| Kwizera^23^ | Uganda | Outpatient | 36 | 55 | 38 | 0 |
| Lechiile^24^ | Botswana | In & outpatient | 37 | 49.6 | 32 (CrAg+); 56 (CrAg-) | NR |
| Letang^25^ | Tanzania | Outpatient | 38 | 60 | 71 | 0 |
| Liechty^26^ | Uganda | Outpatient | 38 | 70.6 | 50 | 1.0 |
| Longley^27^ | South Africa | Outpatient | 36 | 53 | 56 | 0 |
| Luzinda^28^ | Lesotho | Outpatient | NR | NR | NR | NR |
| MSF DRC | DRC | Inpatient | NR | NR | NR | NR |
| MSF Kenya | Kenya | Outpatient | NR | NR | NR | NR |
| Magambo^29^ | Tanzania | Outpatient | 36 | 57.9 | 97 |  |
| Makadzange^30^ | Zimbabwe | Outpatient | 37 | 43.4 | 32 | 10.3 |
| Mamoojee^31^ | Ethiopia | Inpatient | 40 | 59 | 28 | 0 |
| Mamuye^32^ | Uganda | Inpatient | 37 | 47 | 93 | 64.0 |
| McKenney^33^ | USA | Outpatient | 39 | 46.7 | 51-100 | 55.9 |
| Mendes^34^ | Brazil | . | 41 | NR | 86.5 | NR |
| Meya^35^ | Uganda | Outpatient | NR | NR | NR | 0 |
| Meyer^36^ | Kenya | Outpatient | 34 | 54 | 43 | 0 |
| Mfinanga^37^ | Tanzania & Zambia | Outpatient | 36.3 | 55 | 50-99 | 0 |
| Mhlanga^38^ | South Africa | In & outpatient | NR | NR | 41 (CrAg+); 49 (CrAg-) | NR |
| Micol^39^ | Cambodia | In & outpatient | 35 | 45 | 24 | 0 |
| Morawski^40^ | Uganda | Outpatient | NR | NR | NR | 0 |
| Ndayisenga^41^ | Guinea | Outpatient | 37 | 66 | 28 | 39.3 |
| Ogouyèmi-Hounto^42^ | Cameroon | Outpatient | 40 | 58 | 149 | 81.4 |
| Oladele^43^ | Nigeria | Outpatient | 40 | 57.9 | 160 | 95.3 |
| Osazuwa^44^ | Nigeria | Outpatient | 31-40 | 59 | 51-100 | 0 |
| Oyella^45^ | Uganda | In & outpatient | 32 | 51.8 | 23 | 0 |
| Pac^46^ | Uganda | Outpatient | 36 | 60 | NR | 0 |
| Patel^47^ | UK | In & outpatient | 47 | 45 | 26 | 0 |
| Pongsai^48^ | Thailand | Outpatient | 39 | 38.2 | 62 | 0 |
| Reepalu^49^ | Ethiopia | Outpatient | 35 | 64 | 210 | 0 |
| Rick^50^ | Lesotho | Outpatient | 35 | 46.5 | 50 | 0 |
| Rugemalila^51^ | Tanzania | Outpatient | 39 | 57 | 96 | 44.09 |
| Sawadogo^52^ | Namibia | Outpatient | 38 | 46 | 85 | NR |
| Smith^53^ | Vietnam | Outpatient | NR | NR | 40 | 0 |
| Tassie^54^ | Uganda | In & outpatient | 34 | 60.9 | NR | NR |
| Temfack^55^ | Cameroon | Outpatient | 38 | 70.7 | 46 | 0 |
| Vallabhaneni^56^ | South Africa | Outpatient | 35.2 | 48.2 | NR | 0 |
| Vidal^57^ | Brazil | Inpatient | 38 | 39 | 25 | 74.2 |
| Vu^58^ | Vietnam | Outpatient | 35 | 26.4 | 25 | 0 |

ART= antiretroviral therapy; CrAg= cryptococcal antigen; DRC=Democratic Republic of the Congo; NR=Not reported; UK= United Kingdom; USA = United States of America

1. Ake J, Maswai J, Kiweewa F, et al. Infectious and Noninfectious Multimorbidity Among HIV Clinic Clients in the African Cohort Study. CROI 2015. Abstract 764.

2. Alemu AS, Kempker RR, Tenna A, et al. High prevalence of Cryptococcal antigenemia among HIV-infected patients receiving antiretroviral therapy in Ethiopia. *PLoS One* 2013; **8**(3): e58377.

3. Andama AO, den Boon S, Meya D, et al. Prevalence and outcomes of cryptococcal antigenemia in HIV-seropositive patients hospitalized for suspected tuberculosis in Uganda. *J Acquir Immune Defic Syndr* 2013; **63**(2): 189-94.

4. Anuradha S, H AN, Dewan R, Kaur R, Rajeshwari K. Asymptomatic Cryptococcal Antigenemia in People Living with HIV (PLHIV) with Severe Immunosuppression: Is Routine CrAg Screening Indicated in India? *J Assoc Physicians India* 2017; **65**(4): 14-7.

5. Bedell RA, Anderson ST, van Lettow M, et al. High prevalence of tuberculosis and serious bloodstream infections in ambulatory individuals presenting for antiretroviral therapy in Malawi. *PLoS One* 2012; **7**(6): e39347.

6. Beyene T, Woldeamanuel Y, Asrat D, Ayana G, Boulware DR. Comparison of cryptococcal antigenemia between antiretroviral naive and antiretroviral experienced HIV positive patients at two hospitals in Ethiopia. *PLoS One* 2013; **8**(10): e75585.

7. Chim B, Soeung S, Heng V, Sopheak T, Lynen L, van Griensven J. Integrated cryptococcal antigen screening and pre-emptive treatment prior to initiation of antiretroviral treatment in HIV-infected adults in Cambodia. 7th IAS, Kuala Lumpor, Malaysia. Abstract *AIDS Clin Res 2013, 4:7*.

8. Chipungu C, Veltman JA, Jansen P, et al. Feasibility and Acceptability of Cryptococcal Antigen Screening and Prevalence of Cryptocococcemia in Patients Attending a Resource-Limited HIV/AIDS Clinic in Malawi. *J Int Assoc Provid AIDS Care* 2015; **14**(5): 387-90.

9. Ezeanolue EE, Nwizu C, Greene GS, et al. Brief Report: Geographical Variation in Prevalence of Cryptococcal Antigenemia Among HIV-Infected, Treatment-Naive Patients in Nigeria: A Multicenter Cross-Sectional Study. *J Acquir Immune Defic Syndr* 2016; **73**(1): 117-21.

10. Faini D, Kalinjuma A, Neborak J, et al. Maximizing Detection and Improving Outcomes of Cryptococcosis in Rural Tanzania. CROI 2016. Abstract 760.

11. Frola C, Guelfand L, Blugerman G, et al. Prevalence of cryptococcal infection among advanced HIV patients in Argentina using lateral flow immunoassay. *PLoS One* 2017; **12**(6): e0178721.

12. Ganiem AR, Indrati AR, Wisaksana R, et al. Asymptomatic cryptococcal antigenemia is associated with mortality among HIV-positive patients in Indonesia. *J Int AIDS Soc* 2014; **17**: 18821.

13. Gonzalez F, Paz P, Valencia F, et al. Use of a rapid test for the diagnosis of cryptococcosis in an HIV positive adult population in the city of Popayán, Colombia.

14. Govender NP, Roy M, Mendes JF, Zulu TG, Chiller TM, Karstaedt AS. Evaluation of screening and treatment of cryptococcal antigenaemia among HIV-infected persons in Soweto, South Africa. *HIV Med* 2015; **16**(8): 468-76.

15. Govender N, Sriruttan C, Greene G, et al. Evaluation of reflex laboratory cryptococcal disease screening, South Africa, 2012-2015.

16. Guha S, Mukherjee M, Dutta N, et al. Routine cryptococcal antigen screening before ART initiation: a study from an ART center of Eastern India. . *8th IAS, Vancouver, Canada, 2015*.

17. Hajiabdolbaghi M, Kalantari S, Jamshidi-Makiani M, et al. Prevalence of cryptococcal antigen positivity among HIV infected patient with CD4 cell count less than 100 of Imam Khomeini Hospital, Tehran, Iran. Iranian J Microbiol. 9;2: 119-121. 2017.

18. Jarvis JN, Lawn SD, Vogt M, Bangani N, Wood R, Harrison TS. Screening for cryptococcal antigenemia in patients accessing an antiretroviral treatment program in South Africa. *Clin Infect Dis* 2009; **48**(7): 856-62.

19. Kadam D, Chandanwale A, Bharadwaj R, et al. High prevalence of cryptococcal antigenaemia amongst asymptomatic advanced HIV patients in Pune, India. *Indian J Med Microbiol* 2017; **35**(1): 105-8.

20. Katchanov J, Jefferys L, Tominski D, et al. Cryptococcosis in HIV-infected hospitalized patients in Germany: Evidence for routine antigen testing. *J Infect* 2015; **71**(1): 110-6.

21. Kebede H, Seyoum H, Abebe Y, et al. The invisible killer: a pilot project on cryptococcal meningitis screening, diagnosis and management in high volume hospitals in Ethiopia. IAS 2016. .

22. Kwan CK, Leelawiwat W, Intalapaporn P, et al. Utility of Cryptococcal Antigen Screening and Evolution of Asymptomatic Cryptococcal Antigenemia among HIV-Infected Women Starting Antiretroviral Therapy in Thailand. *J Int Assoc Provid AIDS Care* 2014; **13**(5): 434-7.

23. Kwizera R, Nguna J, Kiragga A, et al. Performance of cryptococcal antigen lateral flow assay using saliva in Ugandans with CD4 <100. *PLoS One* 2014; **9**(7): e103156.

24. Lechiile K, Mitchell H, Mulenga F, et al. Prevalence of advanced HIV disease and cryptococcal infection in Gaborone, Botswana. CROI, 2017. Abstract 740.

25. Letang E, Muller MC, Ntamatungiro AJ, et al. Cryptococcal Antigenemia in Immunocompromised Human Immunodeficiency Virus Patients in Rural Tanzania: A Preventable Cause of Early Mortality. *Open Forum Infect Dis* 2015; **2**(2): ofv046.

26. Liechty CA, Solberg P, Were W, et al. Asymptomatic serum cryptococcal antigenemia and early mortality during antiretroviral therapy in rural Uganda. *Trop Med Int Health* 2007; **12**(8): 929-35.

27. Longley N, Jarvis JN, Meintjes G, et al. Cryptococcal Antigen Screening in Patients Initiating ART in South Africa: A Prospective Cohort Study. *Clin Infect Dis* 2016; **62**(5): 581-7.

28. Luzinda K, Buard V, Damiani I, et al. Cryptococcal antigen screening by lay cadres using a rapid test at the point of care: a feasibility study in rural Lesotho. *8th IAS, Vancouver, Canada, 2015 Abstract TUPED778*.

29. Magambo KA, Kalluvya SE, Kapoor SW, et al. Utility of urine and serum lateral flow assays to determine the prevalence and predictors of cryptococcal antigenemia in HIV-positive outpatients beginning antiretroviral therapy in Mwanza, Tanzania. *J Int AIDS Soc* 2014; **17**: 19040.

30. Makadzange A, Hlupeni A, Boyd K, et al. High prevalence of CNS dissemination with asymptomatic cryptococcal antigenemia. CROI 2017. Abstrac 743. .

31. Mamoojee Y, Shakoor S, Gorton RL, et al. Short Communication: Low seroprevalence of cryptococcal antigenaemia in patients with advanced HIV infection enrolling in an antiretroviral programme in Ghana. *Trop Med Int Health* 2011; **16**(1): 53-6.

32. Mamuye AT, Bornstein E, Temesgen O, Blumberg HM, Kempker RR. Point-of-Care Testing for Cryptococcal Disease Among Hospitalized Human Immunodeficiency Virus-Infected Adults in Ethiopia. *Am J Trop Med Hyg* 2016; **95**(4): 786-92.

33. McKenney J, Bauman S, Neary B, et al. Prevalence, correlates, and outcomes of cryptococcal antigen positivity among patients with AIDS, United States, 1986-2012. *Clin Infect Dis* 2015; **60**(6): 959-65.

34. Mendes R, Negri A, Tsujisaki R, et al. Lateral flow assay in the early diagnosis of cryptococcosis in severely immunosupressed AIDS-patients from the Midwest Region of Brazil. ICCC. .

35. Meya D, Rajasingham R, Nalintya E, Tenforde M, Jarvis JN. Preventing Cryptococcosis-Shifting the Paradigm in the Era of Highly Active Antiretroviral Therapy. *Curr Trop Med Rep* 2015; **2**(2): 81-9.

36. Meyer AC, Kendi CK, Penner JA, et al. The impact of routine cryptococcal antigen screening on survival among HIV-infected individuals with advanced immunosuppression in Kenya. *Trop Med Int Health* 2013; **18**(4): 495-503.

37. Mfinanga S, Chanda D, Kivuyo SL, et al. Cryptococcal meningitis screening and community-based early adherence support in people with advanced HIV infection starting antiretroviral therapy in Tanzania and Zambia: an open-label, randomised controlled trial. *Lancet* 2015; **385**(9983): 2173-82.

38. Mhlanga M, Sriruttan C, Coetzee L, Glencross D, Govender NP. A cross-sectional laboratory survey to determine the prevalence of cryptococcal antigenaemia in South Africa. 6th FIDSSA Congress, KwaZulu Natal, South Africa. Abstract 5271. 2015.

39. Micol R, Lortholary O, Sar B, et al. Prevalence, determinants of positivity, and clinical utility of cryptococcal antigenemia in Cambodian HIV-infected patients. *J Acquir Immune Defic Syndr* 2007; **45**(5): 555-9.

40. Morawski B, Boulware D, Nalintya E, et al. Pre-ART Cryptococcal Antigen Titer Associated With Preemptive Fluconazole Failure. CROI 2016. Abstract 159.

41. Ndayisenga L, Yuma J-D, TiemTore O, et al. Mycobacterium Tuberculosis Lateral Flow Urine Lipoarabinomannan assay (TBLAM) and Cryptococcal Antigen Lateral Flow Assay (CrAg LFA) as screening among patients with advanced HIV-disease in Conakry, Guinea. *IAS, 2017 Abstract TUPEB0375*.

42. Ogouyèmi-Hounto A, Zannou D, Ayihounton G, et al. Prévalence de l’antigénémie cryptococcique et les facteurs associés chez les patients infectés par le VIH à Cotonou au Bénin. *Journal de Mycologie Médicale, 2016 26, 391—397*.

43. Oladele RO, Akanmu AS, Nwosu AO, Ogunsola FT, Richardson MD, Denning DW. Cryptococcal Antigenemia in Nigerian Patients With Advanced Human Immunodeficiency Virus: Influence of Antiretroviral Therapy Adherence. *Open Forum Infect Dis* 2016; **3**(2): ofw055.

44. Osazuwa F, Dirisu JO, Okuonghae PE, Ugbebor O. Screening for cryptococcal antigenemia in anti-retroviral naive AIDS patients in benin city, Nigeria. *Oman Med J* 2012; **27**(3): 228-31.

45. Oyella J, Meya D, Bajunirwe F, Kamya MR. Prevalence and factors associated with cryptococcal antigenemia among severely immunosuppressed HIV-infected adults in Uganda: a cross-sectional study. *J Int AIDS Soc* 2012; **15**(1): 15.

46. Pac L, Horwitz MM, Namutebi AM, et al. Implementation and operational research: Integrated pre-antiretroviral therapy screening and treatment for tuberculosis and cryptococcal antigenemia. *J Acquir Immune Defic Syndr* 2015; **68**(5): e69-76.

47. Patel S, Shin GY, Wijewardana I, et al. The prevalence of cryptococcal antigenemia in newly diagnosed HIV patients in a Southwest London cohort. *J Infect* 2013; **66**(1): 75-9.

48. Pongsai P, Atamasirikul K, Sungkanuparph S. The role of serum cryptococcal antigen screening for the early diagnosis of cryptococcosis in HIV-infected patients with different ranges of CD4 cell counts. *J Infect* 2010; **60**(6): 474-7.

49. Reepalu A, Balcha TT, Yitbarek T, Jarso G, Sturegard E, Bjorkman P. Screening for cryptococcal antigenemia using the lateral flow assay in antiretroviral therapy-naive HIV-positive adults at an Ethiopian hospital clinic. *BMC Res Notes* 2015; **8**: 702.

50. Rick F, Niyibizi AA, Shroufi A, et al. Cryptococcal antigen screening by lay cadres using a rapid test at the point of care: A feasibility study in rural Lesotho. *PLoS One* 2017; **12**(9): e0183656.

51. Rugemalila J, Maro VP, Kapanda G, Ndaro AJ, Jarvis JN. Cryptococcal antigen prevalence in HIV-infected Tanzanians: a cross-sectional study and evaluation of a point-of-care lateral flow assay. *Trop Med Int Health* 2013; **18**(9): 1075-9.

52. Sawadogo S, Makumbi B, Purfield A, et al. Estimated Prevalence of Cryptococcus Antigenemia (CrAg) among HIV-Infected Adults with Advanced Immunosuppression in Namibia Justifies Routine Screening and Preemptive Treatment. *PLoS One* 2016; **11**(10): e0161830.

53. Smith RM, Nguyen TA, Ha HT, et al. Prevalence of cryptococcal antigenemia and cost-effectiveness of a cryptococcal antigen screening program--Vietnam. *PLoS One* 2013; **8**(4): e62213.

54. Tassie JM, Pepper L, Fogg C, et al. Systematic screening of cryptococcal antigenemia in HIV-positive adults in Uganda. *J Acquir Immune Defic Syndr* 2003; **33**(3): 411-2.

55. Temfack E, Kouanfack C, Loyse A, et al. Prevalence of latent cryptococcosis among HIV-infected patients in Cameroon: the ANRS 12312 PreCASA study. ICCC. .

56. Vallabhaneni S, Longley N, Smith M, et al. Implementation and Operational Research: Evaluation of a Public-Sector, Provider-Initiated Cryptococcal Antigen Screening and Treatment Program, Western Cape, South Africa. *J Acquir Immune Defic Syndr* 2016; **72**(2): e37-e42.

57. Vidal JE, Toniolo C, Paulino A, et al. Asymptomatic cryptococcal antigen prevalence detected by lateral flow assay in hospitalised HIV-infected patients in Sao Paulo, Brazil. *Trop Med Int Health* 2016; **21**(12): 1539-44.

58. Vu D, Nguyen K, Nguyen D, et al. Cryptoccal antigen screening among patients with advanced HIV infection in Vietnam. CROI, 2016. Abstract 741.
